# Carbopol Based Hydrogels for ITOPRIDE Hydrochloride Delivery; Synthesis, Characterization and Comparative Assessment with Various Monomers

**DOI:** 10.3390/jfb13040295

**Published:** 2022-12-12

**Authors:** Muhammad Sarfraz, Rabia Iqbal, Kifayat Ullah Khan, Muhammad Usman Minhas

**Affiliations:** 1 College of Pharmacy, Al Ain University, Al Ain Campus, Al Ain 64141, United Arab Emirates; 2 AAU Health and Biomedical Research Center, Al Ain University, Abu Dhabi Campus, Abu Dhabi P.O. Box 112612, United Arab Emirates; 3 College of Pharmacy, University of Sargodha, Sargodha 40100, Punjab, Pakistan; 4 Quaide-e-Azam College of Pharmacy, Sahiwal 57000, Punjab, Pakistan

**Keywords:** hydrogels, carbopol, itopride hydrochloride, controlled release, swelling studies

## Abstract

The objective of the current study was to synthesize and characterize carbopol containing hydrogels with different monomers such as methacrylic acid (MAA), 2-acrylamido-2-methylpropane sulfonic acid (AMPS) and itaconic acid (ITA). Free radical polymerization method was optimized for the preparation of different formulations using *N*,*N*-methylene bis-acrylamide (MBA) as cross linking agent. Different studies were performed to evaluate the effect of different monomers on swelling, drug loading and drug release. Itopride Hydrochloride was used as model drug. FTIR, TGA, DSC and SEM were performed to probe the characteristics of fabricated hydrogels. Swelling studies of different fabricated hydrogels were performed in three pH conditions (1.2, 4.5 & 6.8). Higher swelling was observed at pH 6.8. An in-vitro release study was performed on pH 1.2 and 6.8. The synthesized hydrogels exhibited excellent mechanical strength, higher drug loading, pH sensitive and time dependent release up to 30 h. The excellent mechanical strength and extended drug release of Carbopol-co-poly-MAA-ITA hydrogels make them a potential candidate for controlled delivery of Itopride hydrochloride.

## 1. Introduction

An optimal drug delivery system should be mechanically strong, inert, biocompatible, and acceptable for patients, simple to administer and to remove from the body. Drug administered orally is most convenient and preferred route for drug delivery system to reaches the systemic circulation [1,2]. In the present era, oral controlled drug delivery systems have been gaining much more attention due to improved therapeutic efficacy of developed formulations. A major problem for oral drug delivery systems is the gastric residence time of dosage form. To improve the bioavailability of orally administered drug such type of dosage form should be developed which have the ability to control the drug release as well as prolong gastric residence time.

Hydrogels offer sustained drug delivery systems and overcome the problems associated with short gastric retention time. Hydrogels are three dimensional high-molecular weight networks comprising of a polymeric backbone and a cross-linking agent [3,4]. Hydrogels are employed in various applications in medical and pharmaceutical fields, e.g., wound dressings [5], contact lenses [6], artificial organs, and drug delivery systems [7]. The fabrication of hydrogels involves a number of different monomers and polymers. A wide range of polymers is available which may include natural, synthetic, semi synthetic, biodegradable and non-biodegradable [8,9]. The natural polymers are biodegradable and biocompatible but they are associated with batch-to-batch variations. Synthetic polymers make them an ideal candidate due to their tunable properties [10].

**Figure 1:**
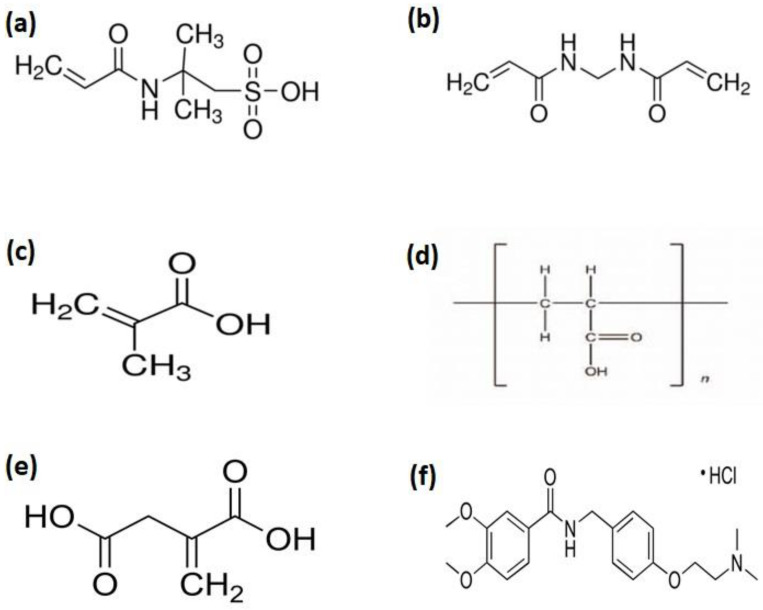
Chemical structure of (**a**) AMPS, (**b**) MBA, (**c**) MAA, (**d**) Carbopol, (**e**) Itaconic acid (ITA), and (**f)** Itopride Hydrochloride.

Carbopol (Figure 1d) is a high molecular weight polymer of acrylic acid [11]. Its uses are widespread in pharmaceutical formulations including gels, emulsions, suspensions, and as thickening and viscosity agents for the purpose of alteration of flow characteristics [12,13,14]. It possesses muco-adhesive properties and the bio-adhesive potential of Carbopol polymers has also been investigated. It is also widely used in ophthalmic, nasal, buccal, intestinal, rectal, vaginal, and topical formulations [15,16].

AMPS (2-Acrylamido-2-methylpropane sulfonic acid) (Figure 1a) is used as a monomer in hydrogel formulations [17]. In AMPS ionizable sulfonic-acid group is present which respond to wide pH range [18,19]. Methacrylic acid (MAA) (Figure 1c) exhibited pH responsive behavior and has the ability to swell in basic pH and collapse in acidic pH [20]. The carboxylic acid group is present in MAA which endorses electrostatic repulsive forces and causes swelling of hydrogel [21]. itaconic acid is prepared by fermentation of carbohydrates, molasses and starch [22]. It possesses high substitution with MAA and acrylic acid. It has two carboxylic acid groups with different pKa value which impart greater hydrophilic characteristics and exhibited good pH sensitivity [23,24]. Moreover, hydrogel mechanical strength increased by co-monomers incorporation which contribute to hydrogen bonding [25].

Itopride hydrochloride (Figure 1f) is a water soluble oral gastric prokinetic agent. It is indicated to treat gastrointestinal motility disorders such as chronic gastritis, gastrointestinal reflux disease, and diabetic gastroparesis [26]. It is an ideal drug for the purpose of developing controlled drug delivery systems because of its short biological half-life (6 h), higher dosing, frequency, and 60% bioavailability [27].

In the present research work, different polymeric networks (hydrogels) have been synthesized. A semi-synthetic polymer (Carbopol) was cross-linked chemically with various monomers, i.e., 2-Acrylamido, 2-methyl propane sulfonic acid (AMPS), methacrylic acid (MAA) and itaconic acid (ITA). *N*,*N*-Methylene bis acrylamide (MBA) was used as cross-linker while Ammonium per oxo disulphate (APS) was employed as reaction initiator. Free radical polymerization was used to fabricate the hydrogels. Itopride Hydrochloride was loaded in the fabricated hydrogels, and we evaluated the effect of the polymer and monomers on swelling, drug loading, and release of hydrogel formulations.

## 2. Materials and Methods

### 2.1. Chemicals

2-acrylamido-2-methylpropane sulfonic acid (AMPS) and Ammonium peroxodisulfate (APS) were obtained from Merck Schuhardt OHG, Hohenbrunn, Germany; *N*,*N*-methylenebisacrylamide (MBA) Merk KGeA, Hohenbrunn, Germany; Methacrylic acid (MAA), itaconic acid, and Carbopol from Sigma-Aldrich, Worcestershire, Dorsett, UK; and Itopride HCL was obtained from Symed Labs Ltd., Telangana, India.

### 2.2. Methodology 

Carbopol based hydrogels were prepared with various monomers by using free radical polymerization method (Figures S1–S3). A viscous polymer solution was formed by dissolving weighed amount of carbapol (0.1 g) in distilled water at room temperature. After this, we dissolved the AMPS (6 g), MBA (0.1 g) and APS (0.2 g) separately in sufficient quantity of distilled water to form clear solutions. The prepared solutions were mixed in order as monomer (AMPS), cross-linker (MBA) and initiator (APS) into polymeric solutions drop wise to obtain a clear homogenous mixture. The final volume (25 g) was made up by adding water. Nitrogen gas was used to purge the reaction mixture in order to remove air or oxygen. The reaction mixture was transferred to pre-labeled glass tubes and placed in a pre-heated water bath at 60 °C for 4 h, after which the temperature was increased to 65 °C for 10 h. The synthesized gels were taken out of the test tubes. The hydrogel was cut using a sharp blade to yield 8 mm discs. The discs were washed with an ethanol:water mixture (30:70) to remove unreacted monomer and catalyst, until there was no change in the pH of the solvent mixture before and after washing the discs. Hydrogel discs were initially dried in laminar flow air for 24 h and then in an oven at 40 °C for one week [28].

A similar method was adopted for preparation of carbopol-co-poly-methacrylic acid hydrogel, and the same concentration was used but in this formulation MAA was used instead of AMPS. We slightly modified the procedure for the preparation of ternary hydrogel containing carbopol (0.1 g) as polymer and two monomers such as methacrylic acid (6 g) and itaconic acid (3 g). In the ternary hydrogel instead of one monomer, two monomer solutions were prepared. itaconic acid was dissolved in ethanol and water mixture (30:70). The APS solution was added to a previously prepared methacrylic acid solution. After this, the mixture of itaconic acid solution was added to the mixture of APS and methacrylic acid, and the rest of the procedure was same as mentioned above.

### 2.3. Characterrization of Fabiracted Hydrogels

#### 2.3.1. FTIR Analysis

FTIR was used to confirm the presence of specific functional groups in polymer (Carbopol), monomers (AMPS, methacrylic acid, itaconic acid), and all fabricated loaded and un-loaded hydrogels. Spectra were obtained by crushing the samples in to the desired sizes and recorded on Attenuated Total Reflectance (ATR) Bruker FTIR (Tensor 27 series, Bremen, Germany).

#### 2.3.2. Thermal Analysis

A Thermo-Gravimetric Analysis (TGA) was performed on TGA modules Q5000 series thermal analysis system (TA Instruments, West Sussex, Chichester, UK). Analysis was carried out over a temperature range of 25–600 °C. The flow rate was adjusted at 20 °C/min.

Differential scanning calorimetry (DSC) was carried out on Q2000 series thermal analysis system (TA Instruments, West Sussex, Chichester, UK). A specific amount of sample (0.5–3 mg) was carried and sealed in an aluminum pan. DSC analysis of all synthesized formulations was performed over a temperature range of 25–400 °C keeping the heating rate at 20 °C/min.

#### 2.3.3. Morphological Analysis (SEM)

The sample was analyzed by Scanning electron microscope (JSm-6940-A, Tokyo, Japan). The sample was cut into pieces, placed on a sample stub, and bounded with carbon tape. The sample was inserted in gold sputtering system. After application of a gold layer, the stub was removed from the gold sputtering system. The sample stub containing the sample was placed on the sample stage of SEM chamber. SEM chamber was evacuated in prior. Sample compartment was closed and photomicrographs were taken to analyze the surface morphology of hydrogels.

### 2.4. In Vitro Studies of Fabiracted Hydrogels

#### 2.4.1. Swelling Experiments

In order to evaluate the swelling behavior and pH response of the prepared hydrogels (Carbopol-co-poly (AMPS) hydrogels, Carbopol-co-poly methacrylic acid hydrogels and Carbopol-co-poly Itaconic acid co-poly methacrylic acid hydrogels), swelling studies were conducted (Figure S4). Buffer solutions of pH 1.2 (0.1 M HCL), pH 4.5 (phosphate buffer), and pH 6.8 (phosphate buffer) were prepared. The dried hydrogel discs were weighed and were immersed in respective buffer solutions of each 100 mL. After an appropriate time interval, the discs were taken out, and dried on bloating paper to get rid of excess solution. All the discs were weighed using electrical analytical balance (Shimadzu, Tokyo, Japan). The discs were re-immersed in the respective solutions after weighing. The study was carried out till the achievement of a constant weight of all hydrogel discs. The following equation was used to calculate the % swelling ratio (SR).

(1) $$\mathrm{SR}\%=\frac{W_{s}-W_{d}}{W_{d}}\times 100\%$$

where ***W_s_*** represents the weight of swelling discs and ***W_d_*** represents dried weight of hydrogel discs.

#### 2.4.2. Sol Gel-Fraction

Sol-gel analysis was carried out using soxhlet extraction technique to determine the extent of chemical reaction [29,30]. The soluble unreacted contents of the polymerization reaction are termed “sol contents”. To evaluate the sol contents, 2 mm size slabs of hydrogels were cut and dried at 55 °C till a constant weight is achieved. Dried slabs were placed in Soxhelt apparatus and extraction was carried out in deionized water for 12 h. The extracted gels were again placed in an oven at 55 °C for drying till the achievement of constant weight. Sol and gel fractions were calculated using Equations (2) and (3) respectively.

(2) $$\mathrm{Gel}\;\%\;=\frac{m_{d-}m_{c}}{m_{c}}\times \;100$$

where ***m_d_*** represents the initial weight of dry gel before extraction and ***m_c_*** represents the weight of the gel after extraction.

*Sol fraction* = 100 − Gel fraction (3)

#### 2.4.3. Drug Loading

Drug loading was performed by previously reported diffusion technique using Itopride Hydrochloride as a model drug. For this purpose 1% drug solution was prepared in a phosphate buffer of pH 6.8. The drug solution was prepared by the addition of drug in parts to the phosphate buffer (pH 6.8) with continuous stirring on a magnetic stirrer. The acidic nature of Itopride hydrochloride lowered the pH of the medium to 6.5 which was increased by addition of NaOH (50%) drop wise. Dried Carbopol based hydrogel discs were immersed in the 100 mL Itopride hydrochloride solution (1%) at room temperature for 48 h till equilibrium weight was achieved using analytical weighing balance. After the drug loading, discs were taken out, washed with distilled water to remove any surface deposition of drug. The discs were then placed in oven at 40 °C for drying. The following relation was used to measure the percent drug loading [29,31].

Amount of drug loaded = W_D_ − W_d_ (4)

Percent drug loading = (W_D_ − W_d_/W_d_) × 100 (5)

where W_D_ represent weight of dried drug loaded discs and W_d_ represents the weight of hydrogel discs before loading.

#### 2.4.4. Loaded Drug Contents (LDC)

The drug loaded contents of prepared Carbopol based hydrogels was determined. A clean and dried pestle and mortar was used to crush the drug loaded hydrogels. The accurately weighed quantity of powdered hydrogels was added to the phosphate buffer (500 mL) of pH 6.8 for 24 h with constant stirring at temperature of 37 ± 0.5 °C. After 24 h centrifugation was carried out at the speed of 3000 RPM (revolutions per minute). The supernatant layer was then separated and filtered using a filter paper of pore size 0.45 µm. Assay of Itopride hydrochloride was performed by UV spectrophotometer at λ_max_ 258 nm. The following formula was used to calculate the LDC (mg/g) of hydrogels.

Loaded drug contents (mg/g) = weight of drug in hydrogel/weight of dried crushed hydrogel(6)

#### 2.4.5. In Vitro Drug Release Studies

In vitro drug release studies were performed at both pH 1.2 and pH 6.8. Dissolution studies were carried out using USP dissolution apparatus II at pH 1.2 and pH 6.8. 900 mL solution of each buffer was used in a flask at a time. The studies were performed at 37 °C ± 0.5 °C at 50 rpm. Samples were collected after specific intervals specified prior. The samples were analyzed at λ_max_ 258 nm using UV visible spectrophotometer 1700 (PharmaSpec Shimadzu, Tokyo, Japan).

## 3. Results and Discussions

### 3.1. FT-IR Spectroscopy

**Figure 2:**
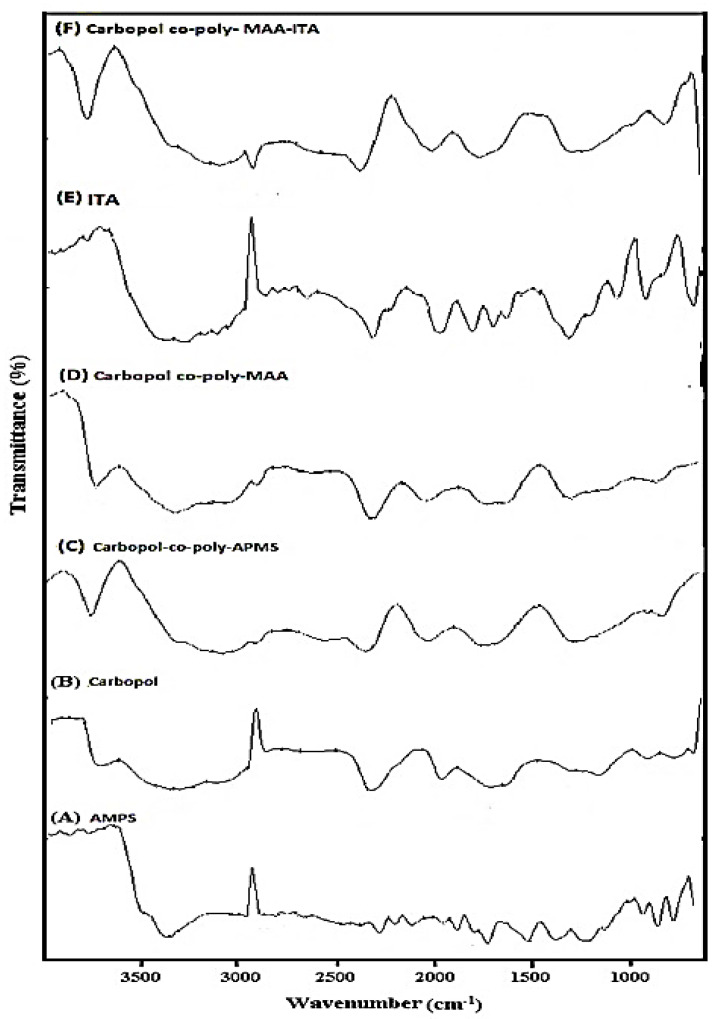
FTIR spectrum of pure (**A**) AMPS, (**B**) Carbopol, (**C**) MBA, (**D**) Carbopol-co-poly APMS Hydrogel, (**E**) Carbopol-co-poly MAA hydrogels, pure ITA, and (**F**) Carbopol-co-poly ITA-MAA hydrogels.

The FTIR spectra of AMPS, Carbopol and Carbopol-co-poly AMPS hydrogels are shown in Figure 2A–C respectively. A characteristic band observed in the range of 3600 to 3100 cm^−1^ was attributed to the –OH and amine N–H symmetrical stretching vibrations. Sharp band at 2924 cm^−1^ was attributed to –C–H stretching vibrations of methylene and methyl groups. A feeble peak present at 2854 cm^−1^ and 1717 cm^−1^ was due to the stretching of –C–C and –C=O respectively while a sharp peak at 1661 cm^−1^ was representing the –N–H bending vibrations. The –C–H bending vibrations of methyl and methylene functional groups is responsible for making various weak peaks in range of 1300–1550 cm^−1^. Principle bands present at 1226 and 1043 cm^−1^ were due to sulfonate functional group (–S=O) and due to stretching vibrations of –C–N. The FTIR spectrum of Carbopol (Figure 2B) indicates a specific peak in the range of 3000–1940 cm^−1^ was due to the stretching vibrations of –O–H group of carboxylic acid and intra-molecular hydrogen bonding. A significant peak present at 1700 cm^−1^ was attributed to the stretching vibrations of carbonyl group (–C=O). Peaks present in the range of 1460–1400 cm^−1^ represented the –C–O & –OH– bending vibration while peaks at 1250–1200 cm^−1^ were assigned to bending of –C–H of methylene. A characteristic peak at 1157 cm^−1^ indicated the stretching of C–C. FTIR spectrum of Carbopol-co-poly AMPS hydrogels, as shown in Figure 2C, indicated a swing to a different pattern in comparison to pure polymers and monomer, which may be indicating the formation of polymeric network structure.

FTIR spectrum of Carbopol-co-poly MAA hydrogels are shown in Figure 2D. FTIR spectrum of MAA indicated the band at 2987 cm^−1^ which was attributed to the presence of methyl asymmetric stretching. The significant peaks present at 1698 & 1635 cm^−1^ were assigned to the presence of C=C functional group and stretching vibration of carboxylic acid. The FTIR spectrum of Carbopol-co-poly methacrylic acid hydrogel (Figure 2D) exhibited peaks which were different from its parent components (carbopol and methacrylic acid). Peak at 3460 cm^−1^, the absorption corresponded to the OH stretching and peak at 1694 cm^−1^, with absorption indicating a carbonyl group, thereby revealing esterification between Carbopol and MAA.

FTIR spectra of pure ITA and Carbopol-co-poly ITA-MAA is depicted in Figure 2E,F. Spectrum of pure ITA was also recorded and the peaks present at 1682.82 cm^−1^, 1624 cm^−1^ & 1435 cm^−1^ were assigned to the plane bending of C=O, C=C and C–O–H respectively. The peak present at 1214.80 cm^−1^ was attributed to the C–O stretching. FTIR spectrum of co-polymeric hydrogel ITA-MAA (Figure 2F) indicated the successful cross-lining of MAA & ITA due to the presence of three prominent peaks. The peak recorded at 3400.49 cm^−1^ was due to stretching of C–O–H of hydroxyl group. The other peaks present at 2917.64 cm^−1^ and 1716 cm^−1^ indicated the CH_2_ stretching and C–O stretching vibration of the carboxylic group.

### 3.2. Thermal Analysis of Fabricated Hydrogels

**Figure 3:**
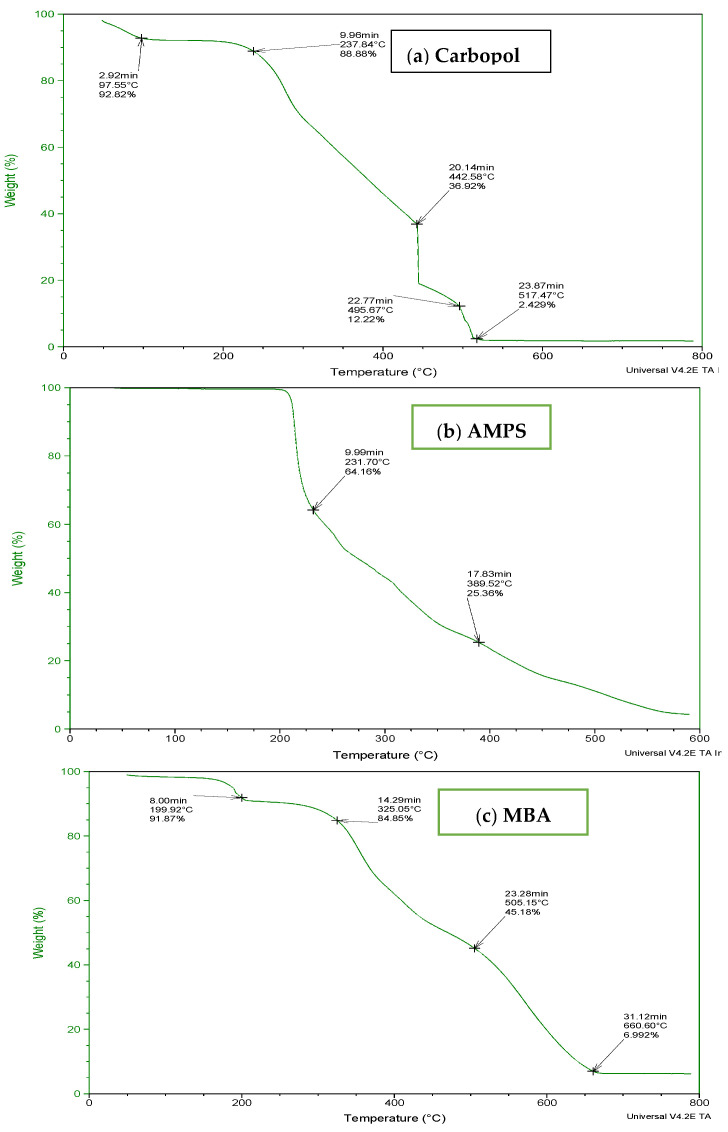

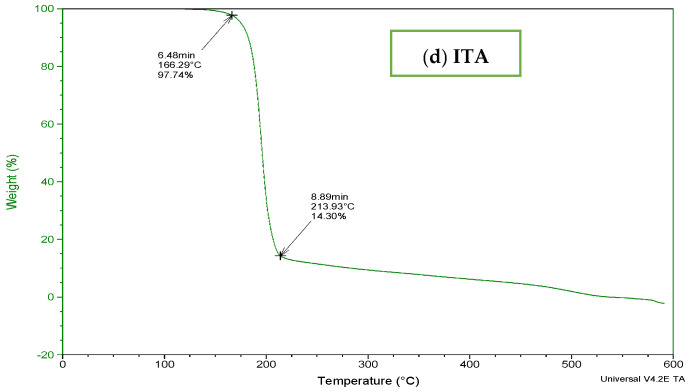
TGA thermogram of (**a**) polymer carbopol, (**b**) monomer AMPS, (**c**) cross-linker MBA and (**d**) ITA.

**Figure 4:**
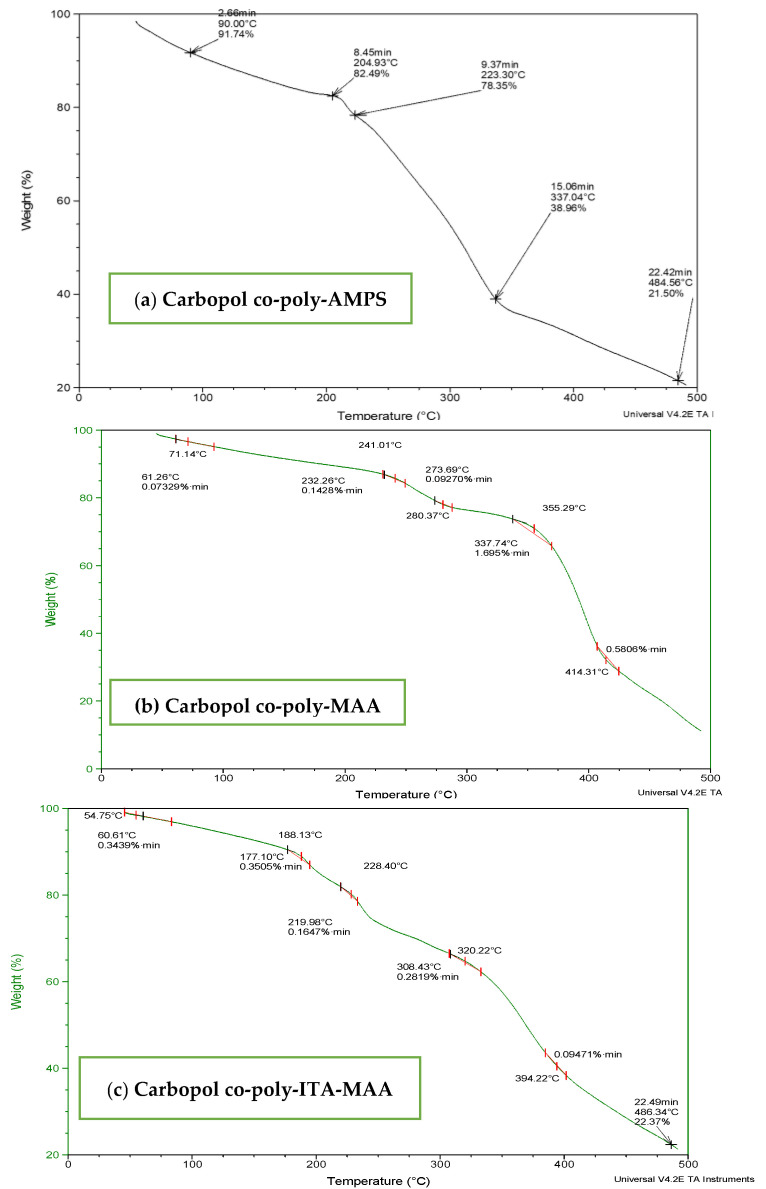
TGA thermogram of (**a**) carbopol-co-poly-AMPS, (**b**) Carbopol co-poly-MAA and (**c**) carbopol co-poly-ITA-MAA hydrogels.

To determine the thermal stability of fabricated hydrogels, TGA and DSC of carbopol, AMPS, MBA, ITA and developed formulations were performed as shown in Figure 3 and Figure 4. It is evident from the thermogram of carbapol that the evaporation of water up to 80 °C accounts for initial loss of weight of Carbopol. The polymeric backbone appears to be stable up to 205 °C. The main polymeric structure undergoes deterioration at 205 °C to 420 °C as shown in Figure 3a. TGA thermogram of AMPS (Figure 3b) indicated an initial loss of weight owing to moisture loss and stability of AMPS up to 250 °C. Decomposition of AMPS structure is visible after 250 °C till the entire deterioration of the polymeric backbone. TGA thermogram of MBA (Figure 3c) indicated water elimination in the range of 30 °C to 275 °C. Degradation starts at 270 °C and there is complete degradation till 630 °C which is visible in the thermogram. The TGA thermogram of itaconic acid in the temperature range of 120 °C to 270 °C exhibits loss of moisture and formation of anhydride ring. The temperature range of 270 °C to 450 °C identifies decarboxylation and carbonization process, as evident from the peak in this range as shown in Figure 3d.

TGA of carbopol-co-poly (AMPS) based hydrogels indicated initial loss of weight above 50 °C and decomposition above 200 °C as shown in Figure 4a. Thermal analysis of cross-linked hydrogels revealed stability above 200 °C which is a reasonable temperature range to be a stable carrier system for delivering drugs. TGA of Carbopol-co-poly-MAA hydrogels was performed as shown in Figure 4b. Thermo-stability was evaluated from thermogram analysis. Initial loss of mass is evident owing to water loss with in temperature range 40 °C to 250 °C. This temperature range corresponds to melting and transitional change of hydrogel. The degradation is evident at a temperature of 350 °C, which make Carbopol-co-poly MAA hydrogels a stable carrier to deliver drugs.

The TGA thermogram of Carbopol-co-poly-ITA-MAA hydrogel indicated an initial loss of water till 175 °C. TGA curve indicated that the ITA hydrogels are stable up-to 250 °C. It indicated that the Carbopol-co-poly itaconic acid-methacrylic acid hydrogels are more stable than individual components as shown in Figure 4c. The decomposition starts at 250 °C till complete degradation.

### 3.3. DSC Analysis of Fabricated Hydrogels

**Figure 5:**
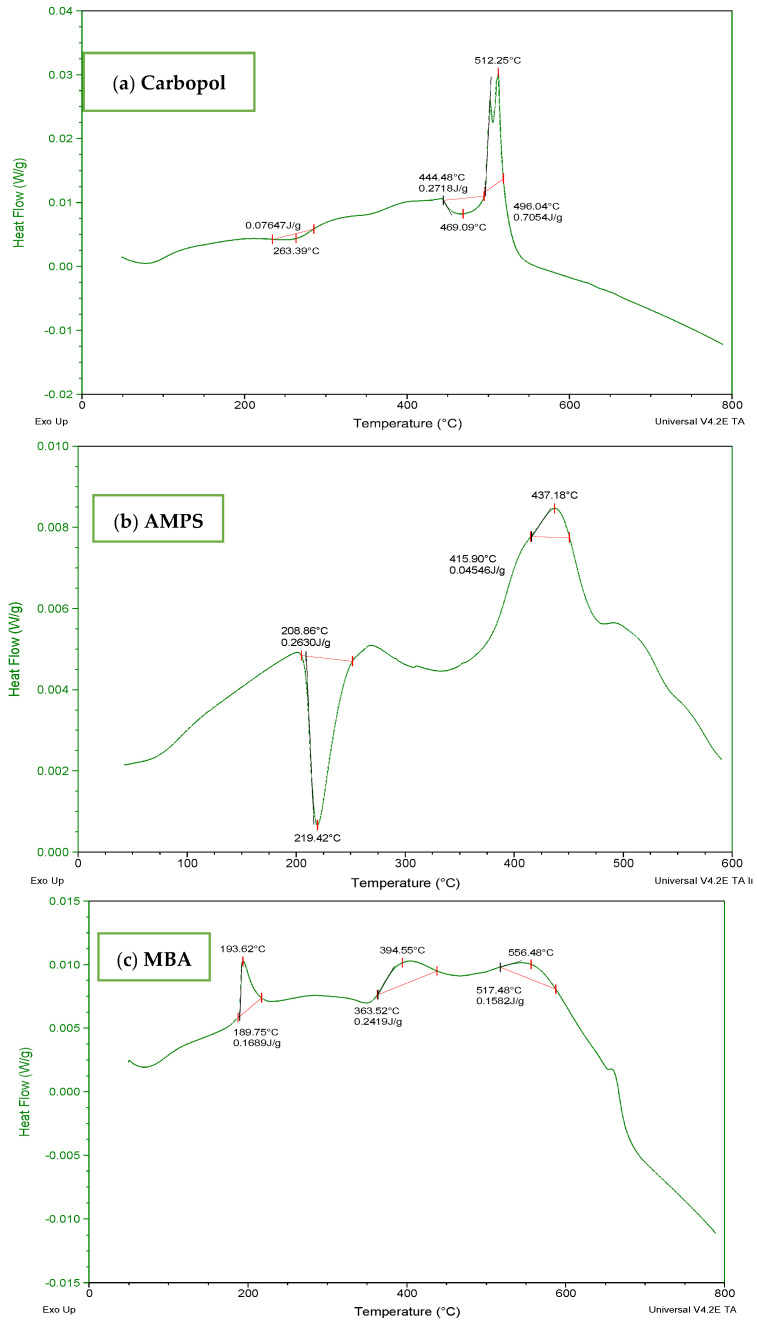

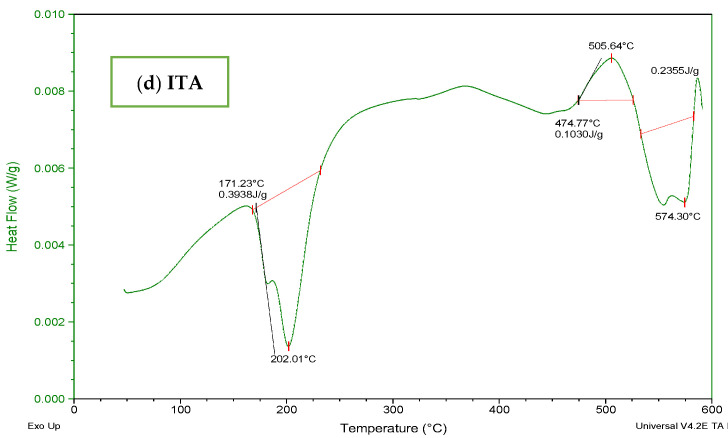
DSC thermogram of (**a**) Carbopol (**b**) AMPS, (**c**) MBA, and (**d**) ITA.

The DSC thermogram of Carbopol (Figure 5a) indicated a wide endothermic peak within the range of 70–300 °C. It depicted melting and denaturation of polymer. There was the presence of a sharp exothermic peak at 500 °C. With the increase in temperature, the polymeric structure deteriorated as indicated by the exothermic response in thermogram. The DSC thermogram of AMPS (Figure 5b) revealed glass transitions and polymeric transitions at 45 °C. At 220 °C the presence of an endothermic peak exhibits melting and polymeric denaturation. With the rise in temperature the polymeric backbone is completely degraded. The DSC thermogram of MBA (Figure 5c) showed loss of water at 50 °C. A major exothermic peak appears at 550 °C and the temperature above 550 °C indicated degradation. The DSC thermogram of ITA (Figure 5d) revealed endothermic reaction at 175 °C and at 200 °C. It is stated in the previous literature that itaconic acid undergoes deterioration by losing water at initial stage and then by forming an anhydride ring [32,33].

**Figure 6:**
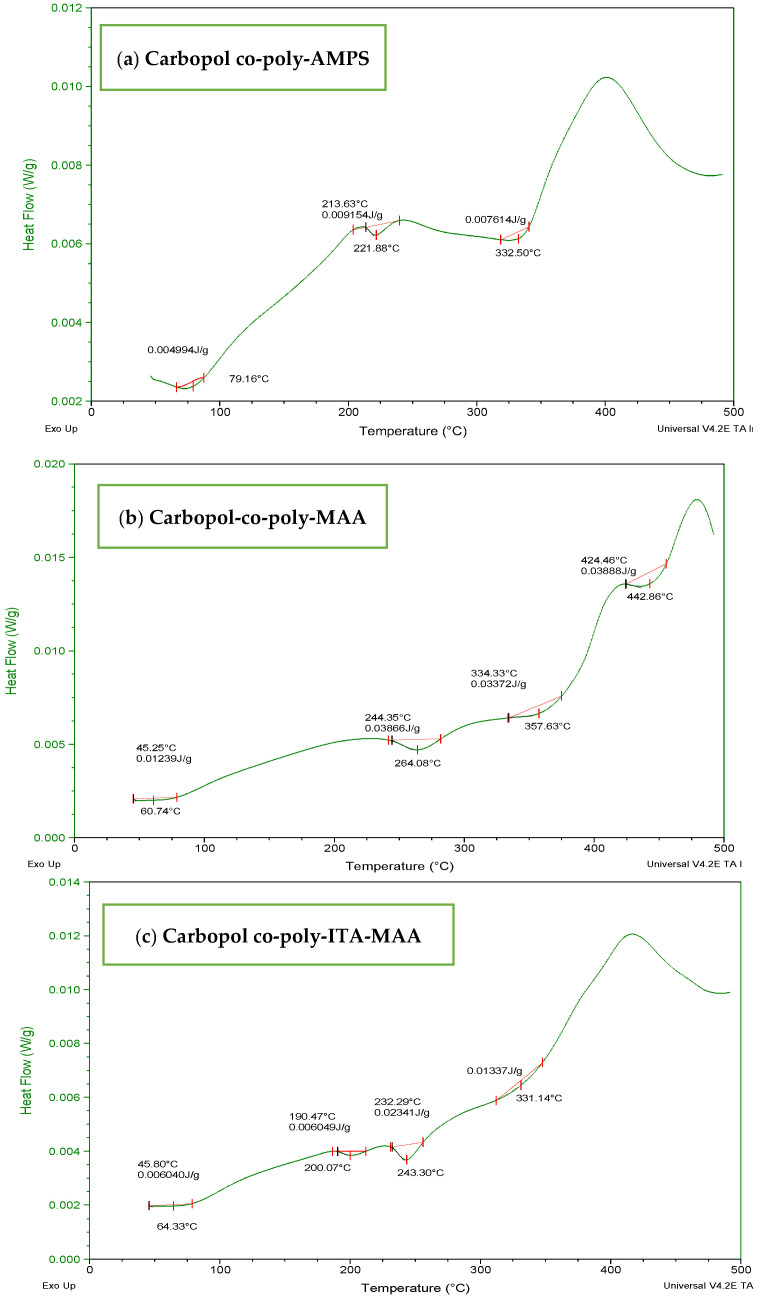
DSC thermogram of (**a**) carbopol-co-poly-AMPS, (**b**) Carbopol co-poly-MAA, and (**c**) Carbopol co-poly-ITA-MAA hydrogels.

The DSC thermogram of Carbopol-co-poly AMPS hydrogels (Figure 6a) showed the stability imparted by cross-linking and initiation of degradation at higher temperature when compared with individual polymers. The endothermic peak at 75 °C may be designated as transition temperature (Tg) and a major endothermic maximum obtained above 330 °C indicate decomposition of the polymeric chain. The DSC thermogram of Carbopol-co-poly MAA hydrogels (Figure 6b) revealed an initial weight loss at 60 °C. The first endothermic peak was observed above 264 °C which indicated the loosening of polymeric chains followed by polymeric transitions and melting. The decomposition curve was obtained at 475 °C. The DSC thermogram of ITA-MAA hydrogels (Figure 6c) indicated the loss of water above 50 °C. The hydrogels appear to be more stable than individual components. They undergo melting and dehydration followed by decomposition. They are stable up to 350 °C after which the major exothermic peak appears, indicating the degradation of the polymeric network.

### 3.4. SEM Analysis of Fabricated Hydrogels

**Figure 7:**
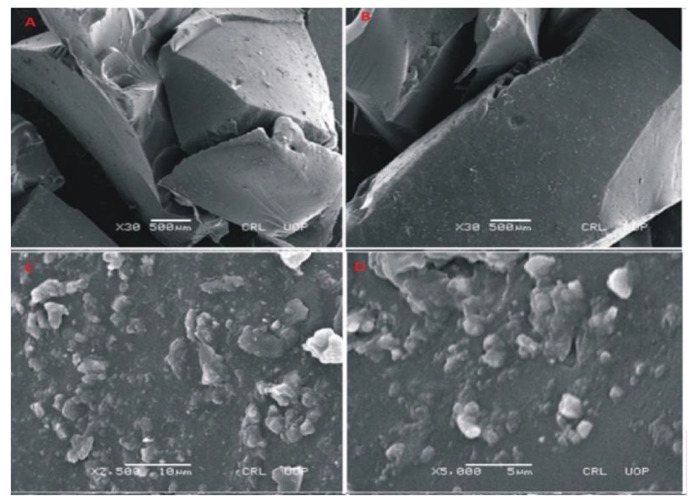
SEM micrographs of cross section (**A**,**B**) and intact surface (**C**,**D**) of Carbopol-co-poly-AMPS hydrogels.

**Figure 8:**
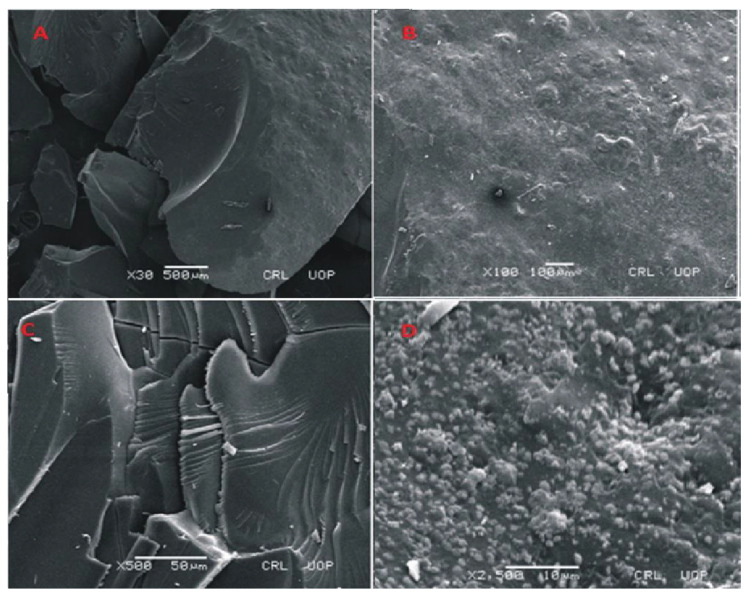
SEM micrographs of cross section (**A**,**B**) and intact surface (**C**,**D**) of Carbopol-co-poly-MAA hydrogels.

**Figure 9:**
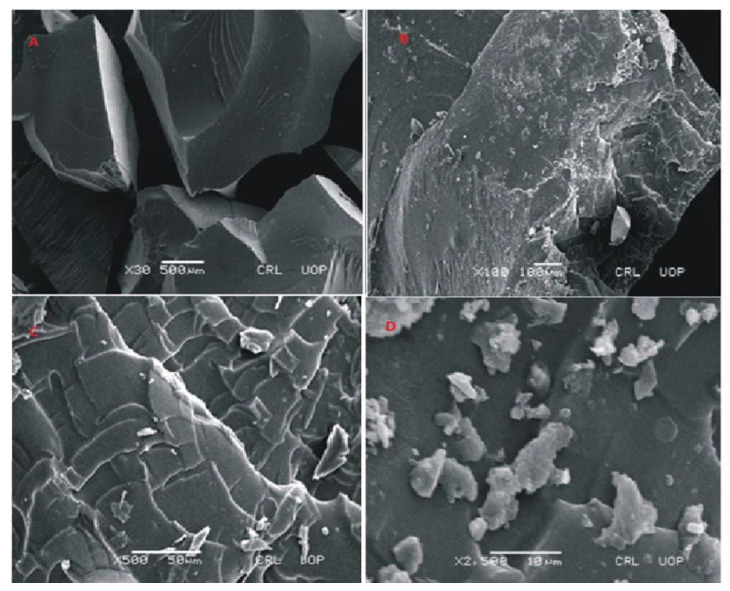
SEM micrographs of cross section (**A**,**B**) and intact surface (**C**,**D**) of Carbopol-co-poly-ITA-MAA hydrogels.

To evaluate the morphology of prepared hydrogels, photomicrographs were obtained at different magnifications using scanning electron microscopy (SEM) as shown in Figure 7, Figure 8 and Figure 9. SEM image of Carbopol co-poly-AMPS as shown in Figure 7 reveals the wavy, rough and porous structure of prepared dried gels at various magnifications. The porous surface of the hydrogels facilitates intake of solvent. These pores provide channels for solvent penetration. The absorbance of solvent creates spaces in the network. This rough and porous surface facilitates drug loading and drug release according to the swelling behavior of the network. In another study it was further confirmed that the presence of AMPS in the formulation is highly responsible for the development of pores [34].

SEM images of Carbopol-co-poly methacrylic acid indicated a rough, scaly and non-porous structure. The non-porous structure limits the penetration of solvent. The presence of a non-porous structure makes network more compact. Lack of pores not only limits the swelling but drug loading and drug release rates are also lower. The presence of scales indicates a brittle structure as well. Fragmentation of the network is also visible. The photomicrographs taken at high magnification also indicate fragmentation of the network. SEM micrographs taken at different magnifications are shown in the Figure 8.

The surface morphology of cross-linked polymeric network of Carbopol-co-poly ITA-MAA acid is shown in Figure 9. The photomicrographs obtained at various resolutions revealed the presence of a rough, scaly, and porous structure. The porous structure of polymeric network facilitates the uptake of water and leads to improved drug entrapment efficiency. It can be easily predicted from the results that the high swelling index is attributed to the presence of porous structure. This porous structure aids in swelling, drug loading, and drug release. It was further reported in a previous study that the water carrying pores in a polymeric network function as channels for the penetration of water molecules and lead to increase contact of water molecules with functional groups on polymeric chain [35]. SEM micrographs also indicate the presence of fissures, scales and fragments. It was reported earlier in a study that the process of drying causes the collapse of the polymeric network and the surfaces become rough [36].

### 3.5. Sol Gel-Fraction

Table 1 presents the results of solgel analysis. Sol-gel analysis was carried out to determine the degree of cross-linking and percentage of un-reactant ingredients, i.e., methacrylic acid (MAA), carbopol, and itaconic acid (ITA) in hydrogel structures. The unreacted components of hydrogels like monomers and cross-linker make up the sol fraction of hydrogel. The solubility of sol fraction of hydrogel in physiological solutions is reported in the wider literature [37,38]. The results of sol gel analysis of present study depicted a negligible sol fraction, consuming most of the reactants, and so consequently stable hydrogels were formed as shown in Table 1. The high gel fraction indicated successful cross-linking in hydrogel structure, as well as productivity of the polymerization technique [39].

### 3.6. Swelling Studies of Fabricated Hydrogels

**Figure 10:**
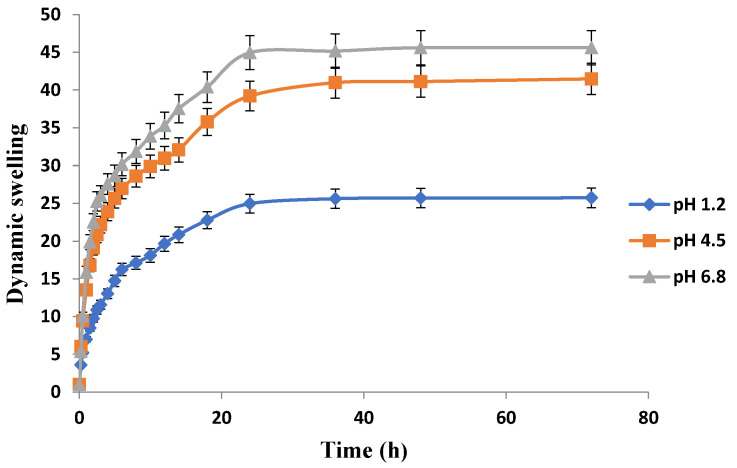
Effect of pH on swelling behavior of Carbopol-co-poly-AMPS hydrogels.

**Figure 11:**
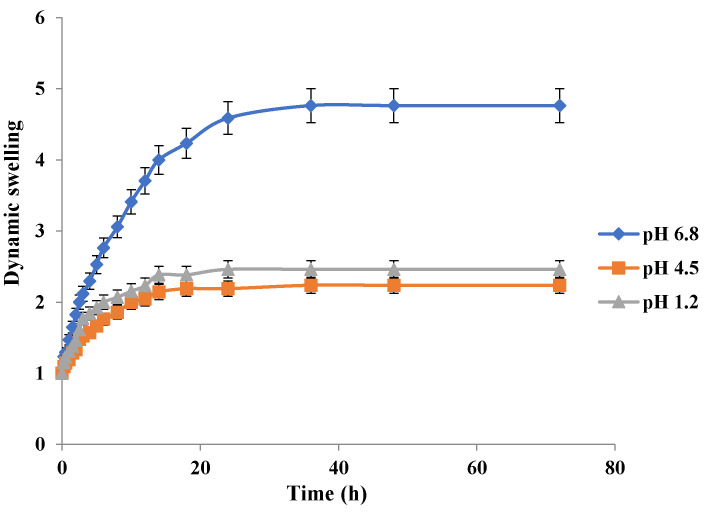
Effect of pH on swelling behavior of Carbopol-co-poly-MAA hydrogels.

**Figure 12:**
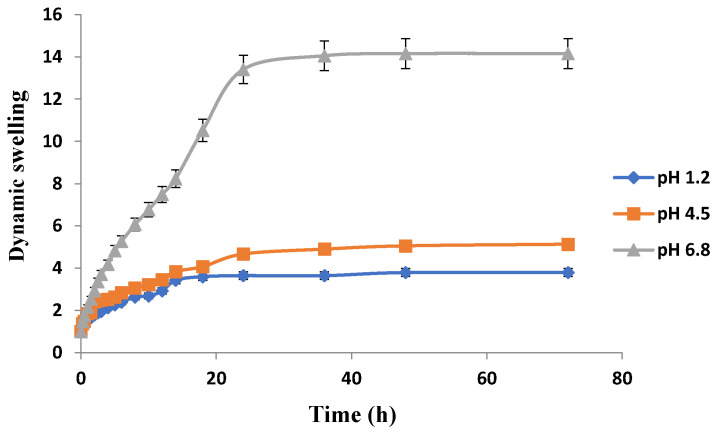
Effect of pH on swelling of Carbopol-co-poly ITA-MAA hydrogels.

The release pattern of the drug-loaded hydrogels at different locations of gastrointestinal tract (GIT) can be best predicted from the swelling behavior of hydrogels in simulated buffers of pH 1.2, 4.5, and & 6.8 as presented in Figure 10, Figure 11 and Figure 12. Swelling behavior of Carbopol-co-poly AMPS hydrogels in simulated fluids with different pH is shown in Figure 10. Carbopol-co-poly AMPS hydrogels exhibited pH independent swelling characteristics and discs showed high swelling index at all pH values. A significant difference in swelling was revealed at pH 1.2 and pH 6.8. However, the difference of swelling index at pH 4.5 and pH 6.8 was insignificant. The swelling index was found to be high at pH 6.8. At 36 h, the swelling index of AMPS based hydrogel in pH 1.2, 4.5 & 6.8 was 25.6, 40.9 and 45.17 respectively. It is therefore clear from the results that there is significant difference in swelling at pH 1.2 and pH 6.8, while there is negligible difference in swelling index between pH 4.5 and pH 6.8. AMPS possesses both ionic and nonionic groups and is a hydrophilic monomer. The presence of strongly ionizable sulfonate groups is responsible for the behavior of AMPS. The dissociation of AMPS, its pH independent swelling, and its high swelling index at a wide range of pH were all reported in another study [40]. It was reported in another study that the entire dissociation of sulfonic acid group and repulsion of ionic charges imparts high swelling to APMS hydrogels. At lower pH values, e.g., 1, similar results were reported [41]. In another study carried out by Liu et al. (1995), AMPS and *N*,*N*-methylenebisacrylamide (MBA) based hydrogels revealed comparable behavior [42]. It was revealed that the hydrogels possessing linear polymers and AMPS exhibited coil expansion, owing to sulfonate groups, upon contact with fluids [43]. A study was conducted by Tong and Liu (1994) on AMPS/DMAA hydrogels. They observed constant swelling rates throughout the pH range and finally stated in the study that the charge density of the AMPS based hydrogels corresponds to the degree of its swelling [44]. S. Ahmed and M. U. Minhas conducted another study and reported pH independent swelling of AMPS [45].

Swelling behavior of Carbopol-co-poly MAA hydrogels in respective simulated media is depicted in Figure 11. MAA contains the carboxylic acid group in its structure which is responsible for its pH sensitivity. The results clearly indicate a significant difference in swelling of MAA hydrogels at pH 6.8 as compared to pH 1.2 and pH 4.5. The swelling index at 36 h at pH 1.2 was 2.38, at pH 4.5 was 2.29, and at pH 6.8 was 4.76. The high swelling index at pH 6.8 is due to the fact that carboxylic groups tend to ionize at a higher pH and a strain of repulsive forces is imparted in the polymeric network. The similar pH dependant swelling effect of MAA has been reported earlier in a study [35]. In another study carried out by S. K. Bajpai and S. Singh in 2006, it was confirmed that MAA hydrogels exhibited pH dependent swelling behavior [46]. Kim and Peppas in 2002 developed poly methacrylic acid-graft-poly ethylene glycol based hydrogels and discovered the cause of the pH dependent swelling behavior. They observed that at pH above that of the the pka values of the polymer (pH ≥ 5), the carboxylic acid groups of MAA get ionized [47].

The swelling behavior of Carbopol-co-poly ITA-MAA acid hydrogels is shown in Figure 12. It is clear that there is significant swelling at pH 6.8. There is no significant difference in swelling rates at pH 1.2 and pH 4.5. At 36 h, swelling index of hydrogels at pH 1.2 was 3.65, at pH 4.5 was 4.90, and at pH 6.8 was 14.05. It was further confirmed from a study conducted by AR Khare and NA Peppas that an increase in pH leads to an increase in dynamic swelling. With increase in pH value above the pka value of the carboxyl groups of itaconic acid, the ionization of the carboxylic groups results in increased swelling [48]. With the increase in pH above the transition pH of the gel, there is immediate dissociation of the complexes, causing an increase in network pore size which results in swelling of co-polymers in the basic environment. The swelling behavior is affected by the external pH which causes the ionization of the carboxylic acid moiety. As the pH rises, the front boundary advances and there is ionization of the carboxyl groups that results in swelling and chain relaxation. Similar results were reported in another study that at high pH, carboxylic acid group gets ionized into carboxylate group, which being hydrophilic in nature imparts electrostatic repulsion and propel the network chain aside. As a result of these electrostatic repulsive forces, hydrogels imbibe more water and swell more with the effect that drug loading and subsequent drug release is greater [35]. pH sensitivity of itaconic acid was reported repeatedly in previous literature as well [49,50,51].

It was observed that all the developed hydrogels swelled to their maximum at pH 6.8. Swelling behavior of Carbopol-co-poly AMPS hydrogels was pH dependent. Carbopol-co-poly methacrylic acid hydrogels showed least swelling and the swelling was pH dependent. Carbopol-co-poly methacrylic acid co-poly itaconic acid hydrogels revealed pH dependent swelling. However, the swelling index of Carbopol-co-poly AMPS hydrogels was much higher when compared to Carbopol-co-poly methacrylic acid hydrogels and Carbopol-co-poly methacrylic acid co-poly itaconic acid hydrogels, as shown in our figures.

### 3.7. In Vitro Drug Release Studies, Absorbency and Loaded Drug Contents of Formulations

**Figure 13:**
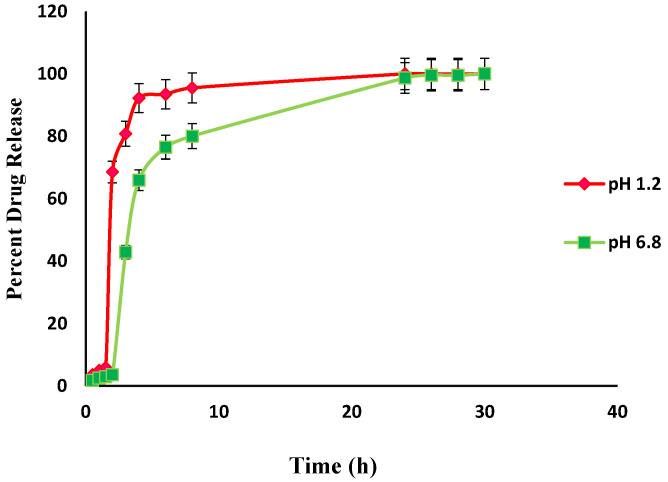
In-vitro drug (Itopride Hcl) release profile from Carbopol co-poly-AMPS hydrogel.

An in-vitro drug release study was performed to determine the controlled release characteristics and drug release behavior of prepared hydrogels, The effect of pH on the release of Itopride hydrochloride from Carbopol-co-poly AMPS hydrogels was determined experimentally (Figure 13). The cumulative release of Itopride hydrochloride from Carbopol-co-poly AMPS based copolymer as a function of time is shown in Figure 13. The discs that have more swelling index were capable of loading more of the drug. Dissolution studies were carried out in simulated buffers of pH 1.2 and pH 6.8. The drug release rates were independent of the pH of the medium in which the hydrogels were present. The hydrogel polymeric network, the interactions of the drug with the polymer, drug solubility, and swelling of the network in dissolution media are some of the factors that govern drug release rates. It has been reported earlier in a study that AMPS based hydrogels exhibit pH independent release. In another study, it was observed that in AMPS based hydrogels, higher percent drug release from polymeric structure is due to highly porous structure and more relaxation of polymeric network, with the consequence that more water uptake is facilitated which leads to more swelling [34].

In the present study, the formulations based on AMPS exhibited pH independent drug release. The drug release rates in pH 1.2 were higher as compared to drug release rates in pH 6.8. This drug release pattern was not consistent with the swelling behavior of same hydrogels during swelling studies. It can be argued that the difference in drug release at different pH was due to the carboxylic groups, as above its pka value carboxylic acid becomes dissociated [52].

**Figure 14:**
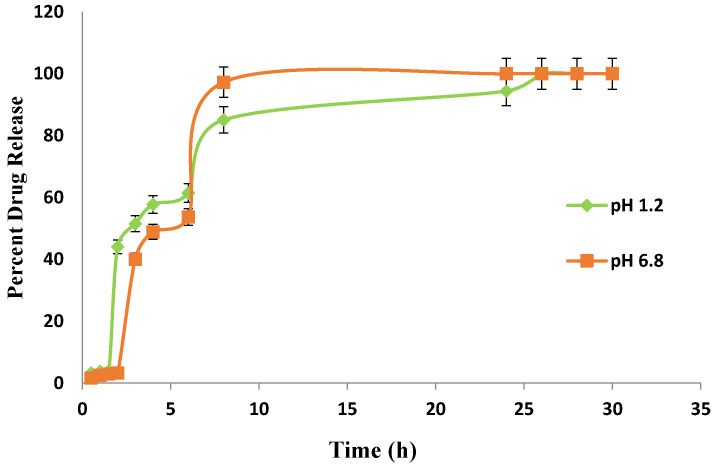
In-vitro drug (Itopride Hcl) release profile from Carbopol co-poly-MAA hydrogel.

In carbopol co-poly-MAA hydrogel (Figure 14), the drug release rates were dependent on the pH of the medium in which the hydrogels were present. It was reported in a study that the presence of hydrophilic groups and carboxylic acid group in MAA is responsible for its pH-sensitive behavior. It was also reported that carboxylic acid group of MAA ionizes at higher pH and employs repulsive forces in polymeric chains [35].

In the present study, the lower swelling index of MAA is because of its greater cross-linked density. MAA forms a dense cross-linked structure that limits the water uptake, and as a result a smaller amount of drug is loaded, which leads to lower release rates. It is also observed that in vitro drug release rates were consistent with the swelling studies performed earlier. In the beginning, the drug release rates were high in pH 1.2 but later on the drug release rates were found to be high in pH 6.8.

**Figure 15:**
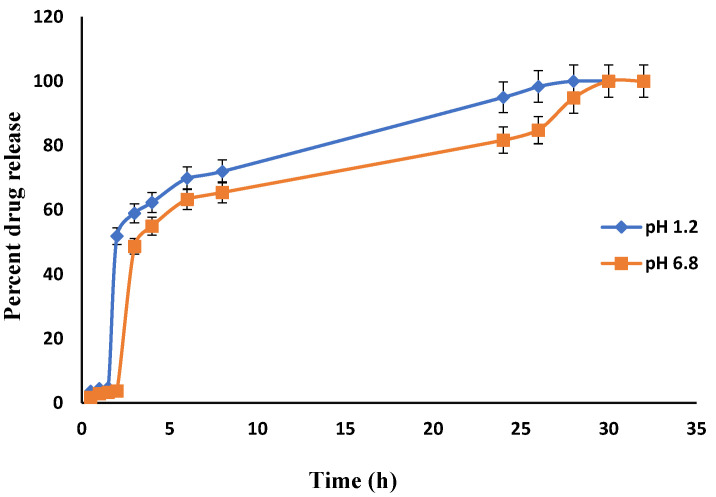
In-vitro drug (Itopride Hcl) release profile from Carbopol co-poly-ITA-MAA hydrogel.

Figure 15 shows the drug release behavior of Carbopol co-poly-ITA-MAA hydrogel. It was observed that the carboxylic group of itaconic acid becomes dissociated into a carboxylate group and exerts electrostatic forces which relaxes the network. The result of electrostatic forces is higher swelling which causes enhanced drug loading and release. Other studies also support the pH-sensitive behavior of itaconic acid [49,50,51].

### 3.8. Comparative Analysis of Drug Release from Fabricated Hydrogels

In the present study, different drug release rates were observed in various developed hydrogels. However, the drug release pattern did not follow the swelling behavior of the hydrogels. It is clear from the above results that the release of Itopride hydrochloride from all hydrogels in pH 1.2 is greater compared to pH 6.8. This is due to the fact that Itopride hydrochloride is basic in nature and highly water soluble. The molecular weight of Itopride hydrochloride is also less, i.e., 394.8 g/mol., so as a result there was more leaching out of drug in acidic media. The results revealed that at pH 1.2, 80% drug was released from Carbopol-co-poly AMPS hydrogels in 4 h, while it took 8 h for Carbopol-co-poly AMPS hydrogels to release the same amount of drug in pH 6.8. It was found that 80% drug was released from Carbopol-co-poly MAA hydrogels in 8 h at both pH 1.2 and pH 6.8. At pH 1.2, 80% drug release was observed from Carbopol-co-poly ITA-MAA hydrogels in 14 h, while the same amount of drug was released from Carbopol-co-poly ITA-MAA hydrogels in 24 h at pH 6.8, as shown in Figure 13, Figure 14 and Figure 15. Loaded drug contents and absorbency of carbopol based hydrogels were evaluated, with the results summarized in Table 1.

## 4. Conclusions

The objectives of the current study have been achieved by synthesizing various cross-linked polymeric matrices, by carrying out their characterization analysis and conducting the drug release studies. The hydrogels were prepared using semi-synthetic biocompatible polymer carbopol. Carbopol-co-poly AMPS hydrogels were fabricated by chemical cross-linking the carbopol with 2-acrylamido-2-methylpropane sulfonic acid (AMPS), methacrylic acid (MAA), and itaconic acid methacrylic combination. The developed polymeric system exhibited excellent mechanical strength, swelling behavior, stability and drug release pattern. Structural analysis confirmed the presence of new chemical bonds indicated the successful cross-linking and development of the system. Thermal studies indicated the stability of developed polymeric hydrogels. SEM micrographs revealed a porous and fluffy structure of hydrogels which facilitates swelling, drug loading, and drug release due to the presence of water channels. The drug release rates from all developed hydrogels were extended over 24 h. The above results revealed that all the synthesized hydrogels can be used successfully for extended release formulations.

## Figures and Tables

**Table 1:** Loaded drug contents (%), Sol-gel fraction (%) and absorbency of hydrogels.

Sr. No.	Formulation	Loaded Drug Contents at pH 6.8 (mg)	
1.	Carbopol-co-poly AMPS hydrogels	974.2 ± 1.25	
2.	Carbopol-co-poly MAA hydrogels	1077.9 ± 1.10	
3.	Carbopol-co-poly ITA-MAA hydrogels	872.42 ± 1.08	
**Sr. No.**	**Formulation**	**Increase in Weight (g)**	**% Absorbency**
1.	Carbopol-co-poly AMPS hydrogels	0.663 − 0.264 = 0.399	83
2.	Carbopol-co-poly MAA hydrogels	0.229 − 0.196 = 0.03	16.8
3.	Carbopol-co-poly ITA-MAA hydrogels	0.558 − 0.439 = 0.12	27.10
**Sr. No.**	**Formulation**	**Sol Fraction (%)**	**Gel Fraction (%)**
1.	Carbopol-co-poly AMPS hydrogels	9.46 ± 1.01	90.54 ± 1.01
2.	Carbopol-co-poly MAA hydrogels	8.35 ± 1.32	91.65 ± 1.32
3.	Carbopol-co-poly ITA-MAA hydrogels	9.04 ± 1.08	90.96 ± 1.08

Values are expressed as Mean ± SD, *n* = 3.

## Data Availability

Not applicable.

## Supplementary Materials

The following supporting information can be downloaded at: https://www.mdpi.com/article/10.3390/jfb13040295/s1, Figure S1. Carbopol-co-poly AMPS hydrogels; Figure S2. Carbopol-co-poly MAA hydrogels; Figure S3 Carbopol-co-poly ITA-MAA hydrogels; Figure S4. Swelling studies of Hydrogels.
