# Supplementary material for: Carbopol Based Hydrogels for ITOPRIDE Hydrochloride Delivery; Synthesis, Characterization and Comparative Assessment with Various Monomers
Source: J Funct Biomater. 2022 Dec 12;13(4):295. doi: 10.3390/jfb13040295 (PMC9785656; doi:10.3390/jfb13040295)
Supplement: Supplementary file 1 [file jfb-13-00295-s001.zip › jfb-2041945-supplementary.pdf]

Journal of
Functional Biomaterials

Article

Carbopol Based Hydrogels for ITOPRIDE Hydrochloride Delivery; Synthesis, Characterization And Comparative Assessment With Various Monomers

Muhammad Sarfraz [1,2], Rabia Iqbal [3], Kifayat Ullah Khan [4] and Muhammad Usman Minhas [3,*]

[1] College of Pharmacy, Al Ain University, Al Ain Campus, Al Ain, United Arab Emirates (UAE); muhammad.sarfraz@aau.ac.ae
[2] AAU Health and Biomedical Research Center, Al Ain University, Abu Dhabi Campus, Abu Dhabi, United Arab Emirates
[3] College of Pharmacy, University of Sargodha, Sargodha (40100), Punjab, Pakistan; drrabeaiqbal@gmail.com
[4] Quaide-e-Azam College of Pharmacy, Sahiwal (57000), Punjab, Pakistan; kifayat.rph@yahoo.com
* Correspondence: us.minhas@hotmail.com or usman.minhas@uos.edu.pk; Tel.: +92-33-1975-0053

Citation: Sarfraz, M.; Iqbal, R.; Khan, K.U.; Minhas, M.U. Carbopol Based Hydrogels for ITOPRIDE Hydrochloride Delivery; Synthesis, Characterization and Comparative Assessment with Various Monomers. *J. Funct. Biomater.* **2022**, *13*, 295. https://doi.org/10.3390/jfb13040295

Academic Editor: Aleksey D. Drozdov

Received: 3 November 2022
Accepted: 9 December 2022
Published: date

Publisher's Note: MDPI stays neutral with regard to jurisdictional claims in published maps and institutional affiliations.

Copyright: © 2022 by the authors. Submitted for possible open access publication under the terms and conditions of the Creative Commons Attribution (CC BY) license (https://creativecommons.org/licenses/by/4.0/).

Physical appearance of Carbopol-co-poly AMPS hydrogels

Stable polymeric networks were synthesized by cross linking of Carbopol with AMPS as a consequence of polymerization.

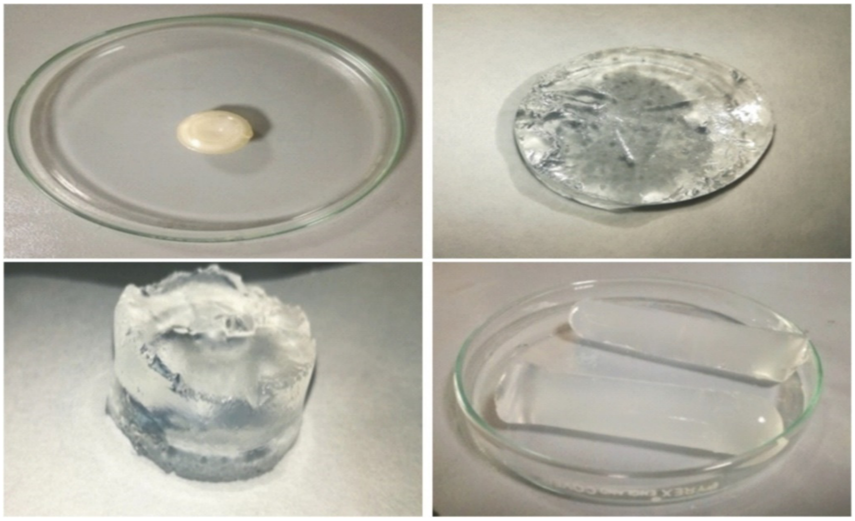

Figure S1. Carbopol-co-poly AMPS hydrogels.

Physical appearance of Methacrylic acid hydrogels

After successful cross-linking and polymerization, stable Carbopol-co-poly MAA hydrogels were formed. The hydrogels obtained were super transparent but after drying they turned less transparent in appearance.

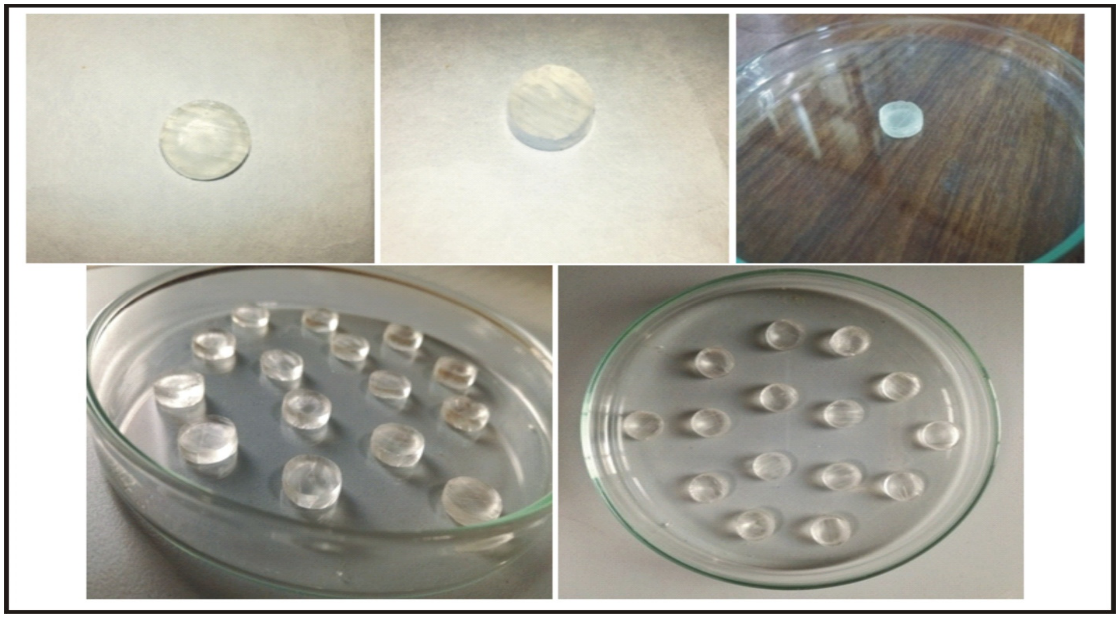

Figure S2. Carbopol-co-poly MAA hydrogels.

Physical appearance of Carbopol-co-poly ITA -MAA acid hydrogels

Stable Carbopol-co-poly ITA co-poly MAA acid hydrogels were synthesized after cross-linking and polymerization. Freshly prepared hydrogels were translucent in appearance but after some time they turned pink brown color.

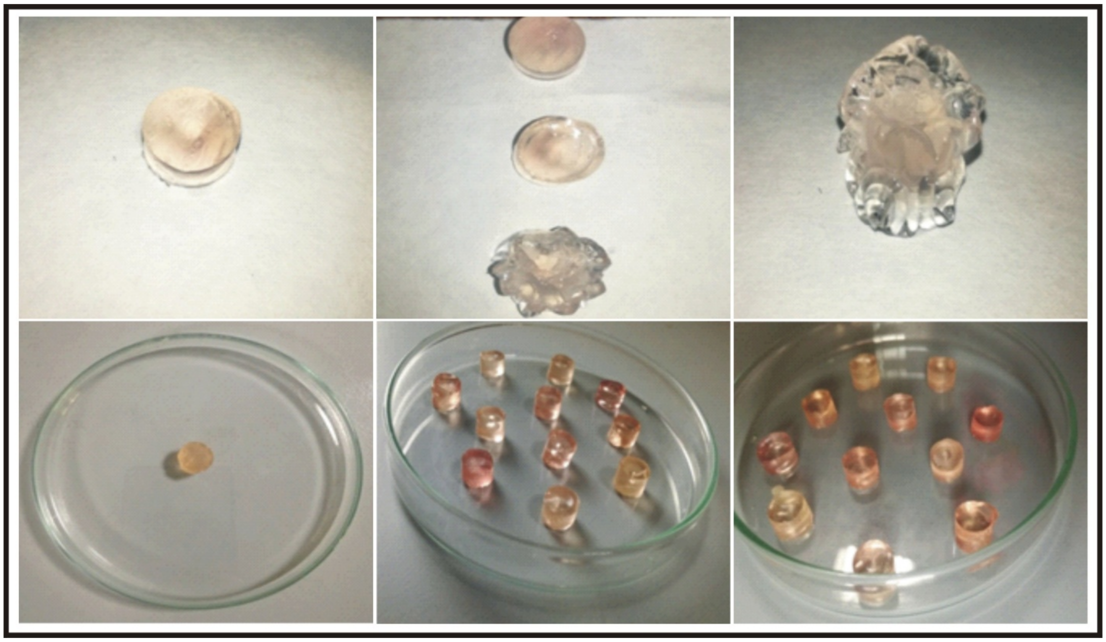

Figure S3. Carbopol-co-poly ITA-MAA hydrogels.

Swelling studies

Swelling experiments were performed in an attempt to find out the effect of pH on the swelling behavior of Carbopol-co-poly (AMPS) hydrogels, Carbopol-co-poly Methacrylic acid hydrogels and Carbopol-co-poly Itaconic acid co-poly Methacrylic acid hydrogels. The experiments were conducted in simulated buffers of pH 1.2, pH 4.5 and pH

6.8. The behavior of the drug loaded hydrogels at different locations of gastrointestinal tract can be predicted from the swelling behavior of hydrogels in simulated buffers of pH 1.2, pH 4.5 and pH 6.8.

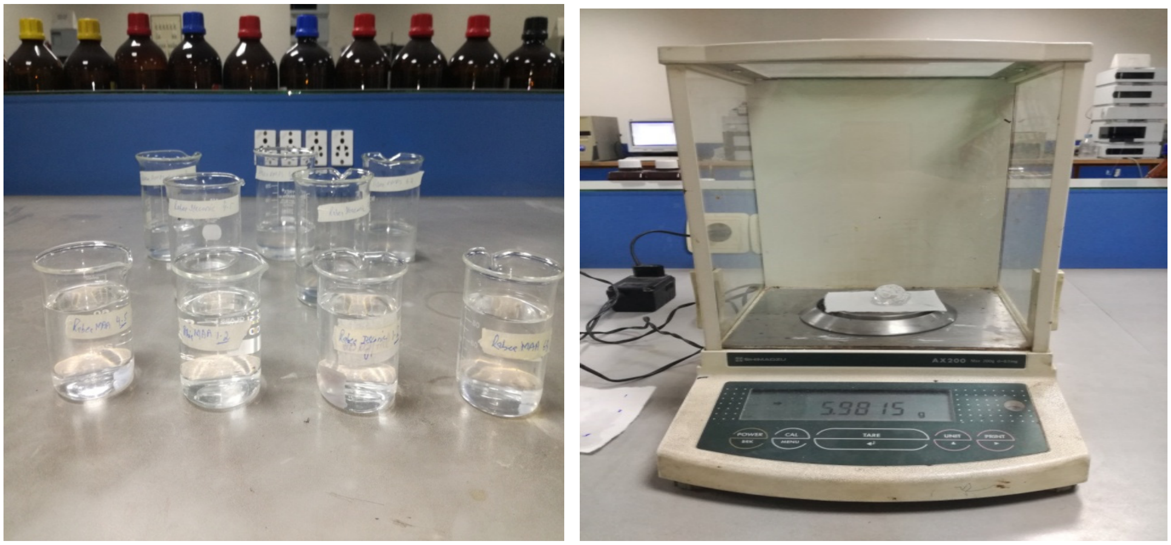

Figure S4. Swelling studies of Hydrogels.
